# Two Different Hemodynamic Responses in ME/CFS Patients with Postural Orthostatic Tachycardia Syndrome During Head-Up Tilt Testing

**DOI:** 10.3390/jcm13247726

**Published:** 2024-12-18

**Authors:** C. (Linda) M. C. van Campen, Peter C. Rowe, Frans C. Visser

**Affiliations:** 1 Stichting CardioZorg, Kraayvel 5, 1171 JE Badhoevedorp, The Netherlands; fransvisser@stichtingcardizorg.nl; 2 Department of Pediatrics, John Hopkins University School of Medicine, Baltimore, MD 21205, USA; prowe@jhmi.edu

**Keywords:** orthostatic intolerance, tilt table testing, POTS (postural orthostatic tachycardia syndrome), stroke volume index, cardiac index, healthy controls, ME/CFS (myalgic encephalomyelitis/chronic fatigue syndrome)

## Abstract

**Introduction**: While the diagnosis of postural orthostatic tachycardia syndrome (POTS) is based on heart rate (HR) and blood pressure (BP) criteria, the pathophysiology of POTS is not fully understood as multiple pathophysiological mechanisms have been recognized. Also, cardiac function, being dependent on preload, afterload, contractility, and HR, has not been properly studied. Preload and contractility changes can be inferred from stroke volume index (SVI) changes during a tilt test. Afterload plays a minor role in POTS as a normal BP response is a prerequisite for POTS. Therefore, we analyzed the HR-SVI relation during a tilt test in myalgic encephalomyelitis (ME/CFS) patients with POTS and compared the data with ME/CFS patients with a normal HR-BP response and with that of healthy controls (HC). **Material and Methods**: In ME/CFS patients with either POTS (*n* = 233) or a normal HR-BP response (*n* = 507) and healthy controls (*n* = 48), we measured SVI (by suprasternal echo), HR, and BP during the tilt. **Results**: In all ME/CFS patients, the decrease in SVI was larger compared to HC. In patients with a normal HR-BP response and in POTS patients with a HR increase between 30–39 bpm, there was an inverse relationship between the HR increase and SVI decrease during the tilt, compatible with increased venous pooling. In POTS patients with a HR increase ≥40 bpm, this inverse relation was lost, and SVI changes were significantly less compared to POTS patients with a HR increase between 30–39 bpm, suggestive of a hyperadrenergic response. **Conclusions**: In ME/CFS patients with POTS, two different hemodynamic profiles can be observed: in patients with a limited HR increase, mainly increased venous pooling is observed, while in patients with a large (≥ 40 bpm) HR increase the data are suggestive of a hyperadrenergic response. These two different profiles may have different therapeutic implications.

## 1. Introduction

Postural orthostatic tachycardia syndrome (POTS) is a common form of orthostatic intolerance. Although symptoms and findings are now clearly defined, its prevalence and pathophysiological mechanisms are not fully understood [1,2]. The syndrome is heterogeneous, and the clinical assessment of patients and treatment are not standardized. Similarly, cardiovascular hemodynamics have not been evaluated extensively. Most studies have found an increased supine HR in POTS patients (see Swai et al. [3] for a review). The increased supine HR has been attributed to vagal impairment [4] and an increased sympathetic drive [5]. Also, in ME/CFS patients with POTS, an increased supine HR was found compared to healthy controls [6,7]. But in these ME/CFS patients, an increased supine HR was also found in patients without POTS [7,8] and was thought to be related to increased catecholamine levels [9], though others [10] did not find this relation. Other plausible explanations are an increased metabolic demand in ME/CFS [11], hypovolemia [12], and a decreased oxygen extraction [13]. Also, conflicting data have been published about stroke volume: studies showed that supine stroke volume (SV) was lower in POTS patients compared to healthy controls [14,15,16] but their data contrast with the results of LaManca et al., Timmers et al., and our data, showing that supine SV/stroke volume index (SVI) in ME/CFS patients was similar to that of healthy controls [7,17,18]. Fu et al. demonstrated that SV reduction was larger in POTS patients compared to healthy controls [15]. In two studies, the decrease in SV was larger in ME/CFS patients than in healthy controls during a tilt test, but no specific SV changes have been reported in ME/CFS patients with POTS.

Although many studies have assessed changes in HR, SV, and cardiac output (CO) during a tilt test [19,20,21,22,23,24], little data are available on the direct relation between HR, SV, and CO changes. In the 1960s, Ross et al. demonstrated that the increase of the HR by atrial pacing resulted in a decrease of SV [25]. The same inverse relation between SV and HR has been previously reported during tilt testing [20]. We previously demonstrated that in the supine, resting state, a similar inverse relation between SVI and HR in ME/CFS patients with either a normal HR and BP response during tilt testing or with POTS was present [7]. Although the slope of the HR-SVI relation in these two patient groups was not different from that of healthy controls, a significant number of the patients had a higher HR for a given SVI, being higher than the upper limit of normal of healthy controls, suggestive of an increased sympathetic activation even at rest in these patients.

In general, during tilt testing, two opposing hemodynamic mechanisms play a role: increased venous pooling resulting in a reduction of SV, and increased catecholamines resulting in increased cardiac contractility, with higher SV. Therefore, we hypothesized that in ME/CFS patients with POTS during the tilt test, the excessive HR increase, compared to ME/CFS patients with a normal HR and BP response and compared to healthy controls, was the combined result of excessive venous pooling with a larger SV reduction, and excessive sympathetic stimulation, leading to an increased contractility and larger stroke volumes. This results in a different HR-SVI relation compared to that of patients with a normal HR-blood pressure response and to that of healthy controls. The different hemodynamic responses may have therapeutic implications, as those with a high HR and hyperadrenergic POTS may benefit from propranolol or ivabradine, while those with predominantly venous pooling may benefit from increased water and salt intake, compression stockings, and medication that increases blood volume, like fludrocortisone.

Therefore, in this study we analyzed the HR-SVI relation in ME/CFS patients with POTS and compared the data with patients with a normal HR-BP response and with that of healthy controls.

## 2. Materials and Methods

We retrospectively reviewed the medical records of all ME/CFS patients who visited the out-patient clinic (Stichting CardioZorg) from October 2012 to February 2022 and who underwent a tilt test. The Stichting CardioZorg is specialized in diagnosing and treating patients with ME/CFS. At the first visit, we determined whether participants satisfied the criteria for ME and CFS [26,27], taking the exclusion criteria into account. No other illnesses were present to explain the symptomatology. For all patients, lab results of the GP were available, and a substantial number of patients visited an internist.

Patients were selected for analysis when both Doppler data of SVI and cardiac index (CI) were available for both the supine position and the upright phase of the tilt test. No drugs influencing HR or BP were used at the time of the tilt testing. A comparison group of healthy controls was recruited from three sources: (a) announcements on ME/CFS patient advocacy websites, (b) posters in the medical clinic’s office building, and (c) healthy acquaintances of the ME/CFS participants. Subjects referred for syncope analysis or for other cardiologic diseases at our clinic were not considered as healthy controls. Disease severity in patients was scored according to the international consensus criteria (ICC), with severity scored as mild, moderate, severe, and very severe [27]. Very severe patients (bedridden patients) were not studied here because they were not able to undergo a tilt test.

The study was carried out in accordance with the Declaration of Helsinki. All ME/CFS participants and healthy controls gave informed, written consent. The study was approved by the medical ethics committee of the Slotervaart Hospital, Amsterdam, for healthy controls P1450 and for ME/CFS patients P1736.

### 2.1. Tilt Test Protocol

Measurements were performed as described previously [28,29]. Briefly, all participants were positioned supine for 20 min before being tilted head-up to 70 degrees. The ME/CFS population is prone to having orthostatic intolerance (see ref [29]) and the tilt test leads to post-exertional malaise [30] in a substantial number of patients. Therefore, tilt duration could be shorter than planned and was determined by the patient’s wellbeing and symptomatology; patients were tilted back upon their own request, to avoid provoking either syncope or worse post-exertional malaise.

As ME/CFS patients had more orthostatic intolerance complaints, and a shorter tilt-duration, data from the mid-tilt acquisition from healthy controls [29] were used for comparison with patient data. Similarly, the mid-tilt images of patients were used when tilt duration was >20 min. The image selection was described previously [31]. As the aim of the tilt test was quantification of orthostatic intolerance, avoiding syncope did not interfere with that goal. HR and systolic and diastolic blood pressures (SBP, DBP) were continuously recorded by finger plethysmography [32,33]. After the test, HR and BP were extracted from the device and imported into an Excel spreadsheet.

The changes in HR and BP during tilt testing were classified according to the consensus statement [34,35,36]: normal HR and BP response (normal HR-BP response), classic orthostatic hypotension (cOH), delayed orthostatic hypotension (dOH), postural orthostatic tachycardia syndrome (POTS), and (near)-syncope. In the present study we only analyzed patients with a normal HR-BP response and patients with POTS, where blood pressures were within normal limits.

### 2.2. Doppler Echocardiographic Measurements

Time velocity integral (VTI) frames were obtained in the resting supine position and at the mid or end of the tilt phase depending on the duration of tilting. The aortic VTI was measured using a continuous wave Doppler pencil probe connected to a Vivid I machine (GE, Hoevelaken, The Netherlands) with the transducer positioned in the suprasternal notch. A maximal Doppler signal was assumed to be the optimal flow alignment. At least 2 frames of 6 **s** were obtained. Echo Doppler recordings were stored digitally. The VTI was measured offline by manual tracing of at least 6 cardiac cycles, using the GE EchoPac post-processing software(Viewpoint 6.12.2) by one operator (C.M.C.v.C.). The outflow tract diameter was manually drawn just below the valve insertion in the parasternal long-axis view of a previously made echocardiogram and the cross-sectional area calculated. As the outflow tract is not circular but ellipsoid, we used the data of Maes et al. [37] to correct for the overestimation by the circular shape of the ellipsoid ventricular outflow tract calculation. In their study, the overestimation of the outflow tract area, using the circular calculation by transthoracic echocardiography, was 24.5%. Therefore, we reduced the outflow tract area by 25%. SVI was calculated from the aortic VTI, multiplied by the corrected aortic valve area, as described previously [38], divided by the body surface area (BSA; DuBois formula), and expressed in mL/m^2^. SVI of the separate cycles were averaged. HR was calculated from the VTI intervals and averaged. CI was calculated by the formula: SVI times HR and expressed in L/min/m^2^.

### 2.3. Statistical Analysis

Data were analyzed using the SPSS statistical package version 29.0.00.0. All continuous data were inspected for normal distribution using Q-Q plots and presented as mean and standard deviation (SD) or as median with the interquartile range (IQR) where appropriate. Nominal data were compared using the Chi-square test (gender and disease severity, 3 × 2 and 3 × 3 tables). Group differences were explored using Welch ANOVA, by the Mann–Whitney U test in case of the comparison of two groups or by the Kruskal–Wallis test in the comparison of three or more groups. Post hoc tests were performed using the Tukey or Dunn test. In this study we categorized the supine HR and HR increase from baseline to end-tilt in cohorts of increments of 5 bpm. A linear regression analysis was performed between the cohorts of supine HR data versus supine SVI and CI data, and between the increase in HR during the tilt versus the decrease in SVI and CI. Due to the large number of comparisons, to reduce type I errors, we choose a conservative *p*-value of <0.01 to be statistically significant. We separately analyzed the data of the suprasternal Doppler to measure stroke volumes and cardiac output, and of the CBF data. The intraclass correlation coefficient (ICC) to assess intra-observer variation of the VTI (Doppler flow velocities) determination was 0.98 and the ICC for inter-observer variation was 0.99 [39]. We also assessed the ICC for the carotid and vertebral artery flows in healthy volunteers. The ICC’s for intra-observer variation of the ICA flow velocity, diameter and flow were 0.99, 0.78 and 0.86, respectively. For the VA, these values were 0.97, 0.91 and 0.92, respectively. The ICC’s for inter-observer variation of the ICA flow velocity, diameter and flow were 0.99, 0.82 and 0.87, respectively. For the VA these values were 0.97, 0.80 and 0.91, respectively [28].

## 3. Results

Of the initially reviewed medical records of 1269 ME/CFS patients, 44 patients were excluded because of having no hemodynamic echo Doppler data during the standing phase of the tilt test or because of insufficient quality. One hundred and eighteen studies were excluded because more than one study was available for a given patient; in these patients only the first test was used. A total of 45 patients were excluded because of having no diagnosis of ME/CFS, 48 due to HR or BP influencing medication, 29 due to lung medication with sympathomimetics, 47 because of an age below 18 years, and 22 because of a BMI > 40. Another 176 patients with orthostatic hypotension or (pre)syncope during tilt testing were excluded. This left 740 ME/CFS patients for analysis. From this group, 507 ME/CFS patients had no tachycardia or hypotension on tilt testing (normal HR-BP response) and 233 ME/CFS patients had POTS as the hemodynamic outcome of the tilt test. From 58 healthy controls, hemodynamic data of 48 were complete and of sufficient quality to be analyzed. Supine VTI image acquisition started at 2.5 (1.2) min before onset of the tilt, and image acquisition lasted 0.6 (0.2) min, without significant differences between the three groups. In POTS patients, end-tilt image acquisition started at 8.9 (2.3) min, in patients with a normal HR-BP at 14.5 (2.5) min, and in HV patients at 15.2 (2.7) min. These differences in the start of end-tilt image acquisition were significantly different between the three groups: *p* < 0.001. Image acquisition lasted 0.6 (0.2) min in POTS patients, 0.7 (0.4) min in normal HR-BP patients, and 0.7 (0.3) min in HV patients. The differences were non-significant.

Table 1 shows the baseline characteristics of ME/CFS patients with POTS or with a normal HR-BP response and of the healthy controls, all of whom had a normal HR-BP response. Patients with POTS were significantly younger and showed a significantly lower BMI compared to the patients with a normal HR-BP response. Disease duration was significantly shorter in POTS patients. Also, POTS patients were more affected by the disease than patients with a normal HR-BP response, as evidenced by a significantly larger percentage of patients with severe disease, and a lower percentage of mild disease.

Table 2 shows the hemodynamic results of the tilt test in ME/CFS patients with POTS or with a normal HR-BP response and in healthy controls. Supine HR was highest in POTS patients and lowest in healthy controls (all three groups significantly different). By definition, end-tilt HR and the HR increase (end-tilt minus supine) were significantly higher in POTS patients than in patients and healthy controls with a normal HR-BP response. Supine SVI was not significantly different between the three groups, but end-tilt SVI was lowest in POTS patients and highest in healthy controls (all three groups significantly different). Consequently, the decrease in SVI was highest in POTS patients and lowest in healthy controls. Supine CI was highest in POTS patients and lowest in healthy controls; the differences were significant between the three groups. End-tilt CI was lowest in patients with a normal HR-BP response; the difference between POTS patients and healthy controls was not significant. The decrease in CI at end-tilt was largest in the patients with a normal HR-BP response, and lowest in healthy controls. The differences in the CI reduction between the three groups were significant. Supine and end-tilt SBP were significantly higher in the patients with a normal HR-BP response compared to the POTS patients. Supine DBP were not different between the two patient groups and healthy controls, while end-tilt DBP were higher in the two patient groups compared to HC.

**Figure 1:**
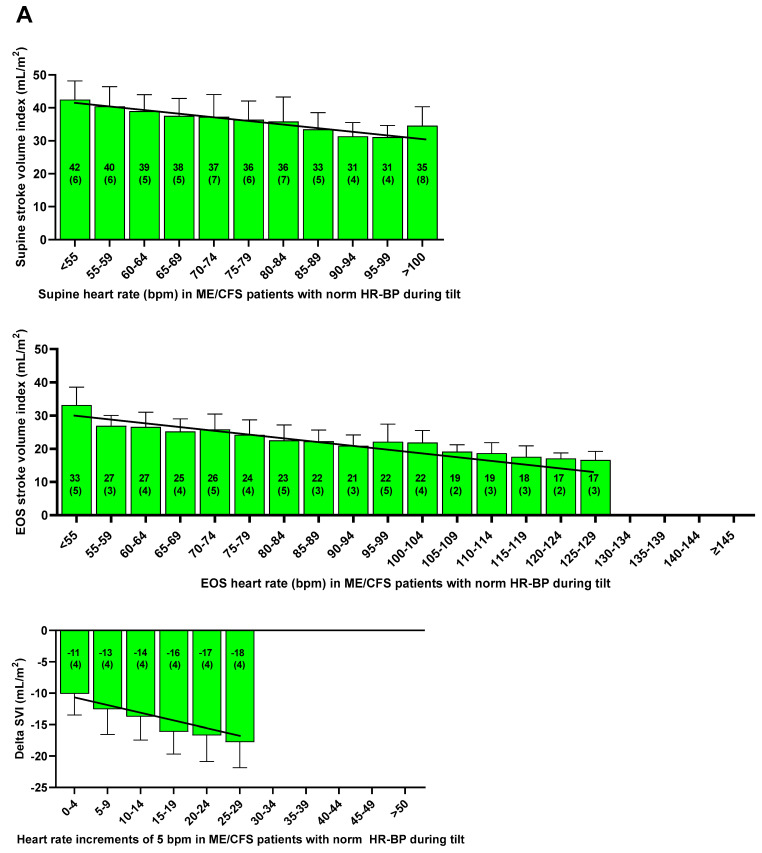

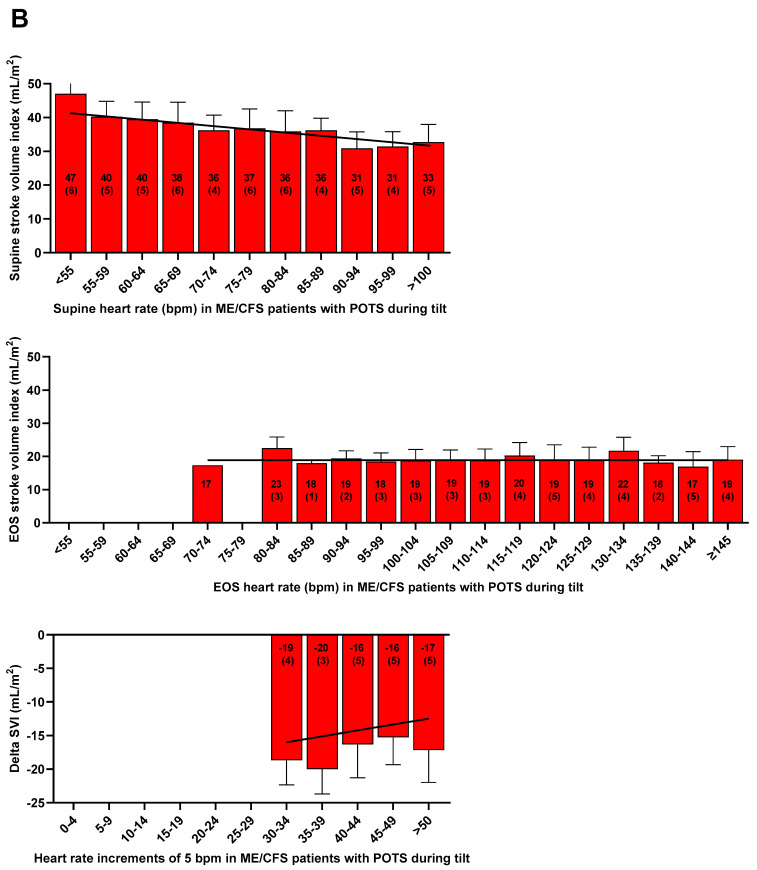

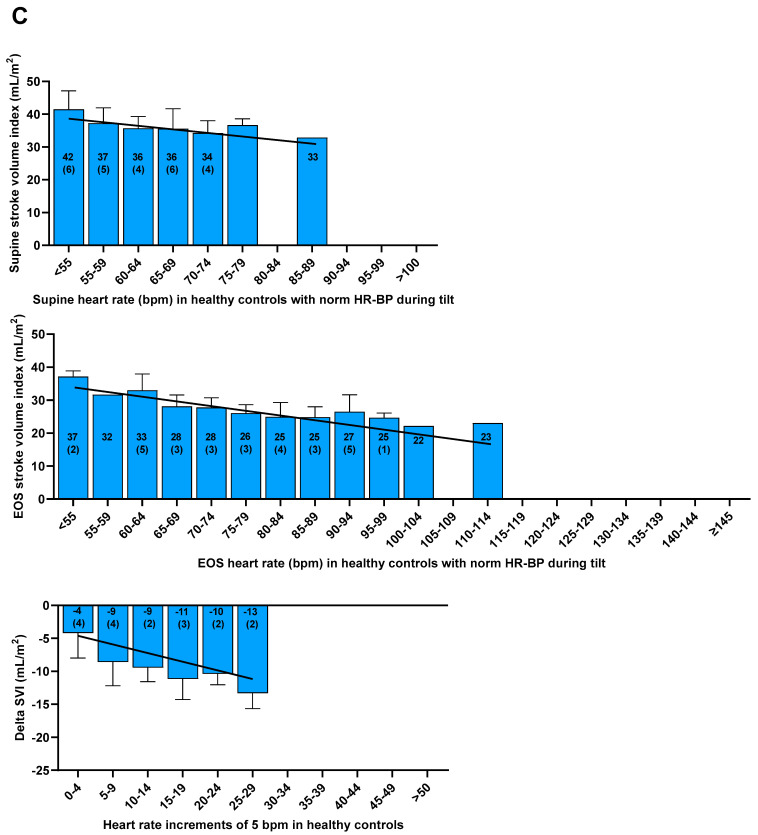
Supine, end-tilt, and delta stroke volume index (SVI) per heart rate cohorts of 5 bpm in ME/CFS patients, with a normal HR and BP response (**A**), with POTS (**B**) and in healthy controls (**C**). Heart rate cohorts of 5 bpm; ME/CFS: myalgic encephalomyelitis/chronic fatigue syndrome; SVI: stroke volume index; red bars ME/CFS patients with postural orthostatic tachycardia syndrome; green bars: ME/CFS patients with a normal HR and BP response; blue bars: healthy controls; delta: supine minus end-tilt data. BP: blood pressure; HR: heart rate; POTS: postural orthostatic tachycardia syndrome; EOS: end of study.

Figure 1 shows the inverse relation between the cohorts of supine HR versus the supine SVI in both patient groups and healthy controls. In both patients and in healthy controls, regression analysis showed that a higher supine HR was significantly associated with a lower supine SVI. Also shown are the end-tilt data of the three groups: in POTS patients, no significant relation between the end-tilt HR and the end-tilt SVI was found, and the slope was not different from zero, in contrast in patients with a normal HR-BP response during the tilt and in healthy controls. In these patients and in HC, a higher end-tilt HR was significantly associated with a lower end-tilt SVI. The bottom three figures show the absolute reduction at end-tilt of SVI in POTS patients, ME/CFS patients with a normal HR-BP response, and healthy controls. In patients with a normal HR-BP response and in healthy controls, a significant inverse relation was found between the HR increase and SVI decrease. In contrast, in POTS patients, there was a positive relation between the increasing HR cohorts and the SVI change: higher HR cohorts were significantly related to lower SVI reductions.

Table 3 shows the data of the regression lines between HR and SVI of the three groups. Supine SVI showed a significant relation with supine HR in all three groups. At end-tilt in POTS patients, no relation between SVI and HR was found, whereas in patients with a normal HR-BP response and in HC, the relationship was significant. In all three groups, the change in SVI vs. the change in HR was significant.

Inspection of Figure 1 shows the first two HR increase cohorts (with HR increases between 30 and 40 bpm) followed the trend of the patients with a normal HR-BP response while the cohorts above 40 bpm of POTS patients SVI reductions were less than of the patients with the first two HR cohorts. These differences in POTS patients are further explored in Table 4. Baseline characteristics like gender, age, length, weight, BSA, BMI, disease duration, and disease severity were not different between the two groups (data not shown).

Table 4 shows the hemodynamic data of the tilt test. Supine HR of the two POTS groups were similar. End-tilt HR was significantly higher in the POTS group with an end-tilt HR ≥ 40 bpm. By definition, the increases in HR of the two groups were significantly different. Supine SVI of the two groups were similar. End-tilt SVI in POTS patients with a HR increase between 30–39 bpm was significantly lower and the SVI reduction significantly larger compared to POTS patients with a HR increase ≥ 40 bpm. As the result of the lower HR increase and larger SVI decrease during the tilt in POTS patients with a HR increase between 30 and 39 bpm compared to POTS patients with a HR increase ≥ 40 bpm, the end-tilt CI was significantly lower, and the CI reduction was significantly larger in the former group. Supine and end-tilt SBP and DBP were not significantly different between the two groups.

**Figure 2:**
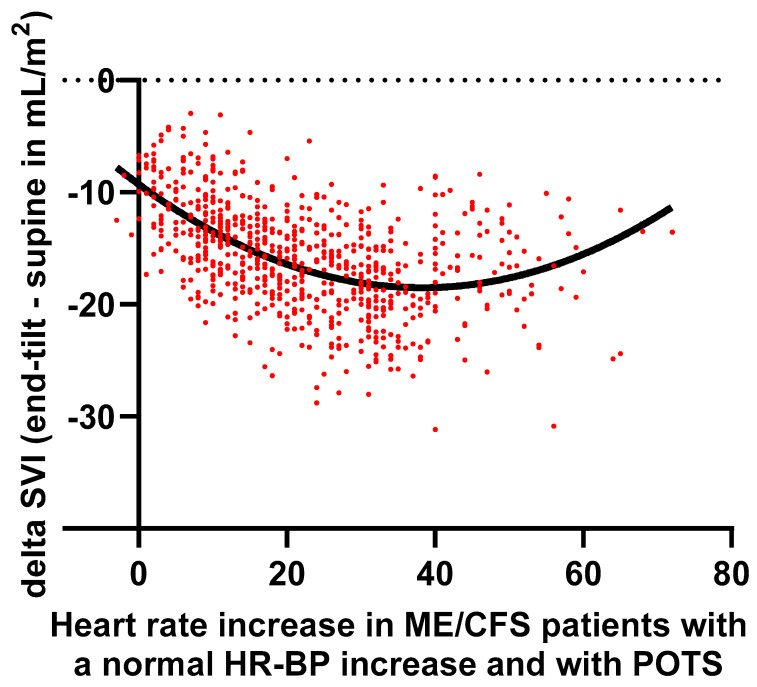
Delta stroke volume index (SVI) versus HR increase in ME/CFS patients with a normal HR and BP (heart rate and blood pressure) response and with POTS using a second order polynomial fit. ME/CFS: myalgic encephalomyelitis/chronic fatigue syndrome; POTS: postural orthostatic tachycardia syndrome; SVI: stroke volume index.

Figure 2 shows the relation between the HR increase and changes in SVI in individual patients with a normal HR-BP response, together with the data in POTS patients. A second order polynomial fit was used. The trough of the polynomial fit was around 40 bpm increase, in line with Figure 1.

## 4. Discussion

While the diagnosis of POTS is based on a clinical definition, the epidemiology and pathophysiology of POTS are not fully understood [40]. In the expert consensus on POTS of the NIH, the authors recognized eight possible mechanisms leading to POTS [2]: hypovolemia, deconditioning, inflammation such as from mast cell activation syndrome (MCAS), excessive central sympathetic activation, auto-antibodies activating cardiac receptors or inhibiting vascular receptors, small fiber neuropathy, and hEDS with connective tissue laxity. Cardiac function (cardiac output) is dependent on the HR, contractility, preload, and afterload. All the aforementioned mechanisms may influence cardiac function in POTS patients.

In the present study, HR, SVI, and CI before and at the end of the tilt test were presented in a large ME/CFS patient population with POTS, and these data were compared with the data of ME/CFS patients with a normal HR and BP response during the tilt and with healthy controls. At rest, in the supine position, we observed a higher HR in POTS patients than in patients with a normal HR-BP response during the tilt and in healthy controls. Our data agree with most previous studies, as they have also found an increased HR at rest in POTS patients (see Swai et al. [3] for a review). The increased supine HR in POTS has been explained by vagal impairment [4], and an increased sympathetic drive to the sinus node [5]. For example, Garland et al. showed that in the supine position, HR and plasma concentrations of norepinephrine, epinephrine, and dopamine were higher in patients with POTS compared with the healthy controls [41]. However, there is heterogeneity in the supine HR of POTS patients. Yoshida et al. recognized two different groups of adolescents with POTS: those with a relatively low supine HR and those with a relatively high HR [42]. Those with a normal supine HR had normal autonomic control of the heart while those with a high supine heart rate had signs of vagal depression. Garland et al. found differences in HR levels of patients with a normal versus high norepinephrine levels [41]. Stewart and Montgomery found differences in supine HR between POTS patients with a low, normal, and high supine CI [43]. Similarly, ME/CFS patients have been observed to have a higher supine HR. Like the POTS studies, these ME/CFS patients may have higher catecholamine levels than healthy controls with higher HR and increased contractility/higher CI [44]. Higher catecholamines in ME/CFS patients have been found by Kristiansen et al. [9] and by Sulheim et al. [45]. However, other groups found no differences in catecholamine levels of CFS patients versus healthy controls in the supine position [6,10]. Other causes of this high resting HR/CI are possibly related to an increased metabolic demand [11], a systemic hypovolemia [12,46], or a decreased oxygen extraction in patients [13,47].

Not only ME/CFS patients with POTS, but also ME/CFS patients with a normal HR-BP response during the tilt have higher supine HR compared to the healthy controls, albeit at a lower level than POTS patients. It is likely that the same above-mentioned mechanisms are operative in these normal HR-BP patients, but this needs further investigation.

Finally, the resting heart rate is related to survival, both in healthy individuals and in patients with different cardiovascular diseases [48], where a higher resting HR was associated with an unfavorable prognosis. Therefore, the prognosis in ME/CFS patients in relation to the resting HR needs to be determined.

Table 1 shows that supine SVI was similar between the three groups, and because of the highest supine HR in POTS patients, supine CI was highest of the three groups. Our data contrast the findings of other studies as mentioned in the review of Natelson et al. [16]. For example, Fu et al. and Miwa and Fujita showed that supine stroke volume and cardiac output were lower in POTS patients [15] and in ME/CFS patients [14]. Fu et al. attributed the lower supine cardiac output and stroke volume to deconditioning [15] and demonstrated that exercise training improved supine and tilt HR, as well as cardiac size and mass, and increased blood volume. Unfortunately, the authors did not mention possible improvement in stroke volume and cardiac output after exercise training. Moreover, they could not exclude the potential influence of advising increasing amounts of water and salt intake. On the other hand, others, like in our study, did not find a difference in supine stroke volume [18,49]. The study of Hurwitz et al. is noteworthy because lower SVI were found, but this lower SVI was for more than 90% explained by blood volume deficits [12]. Further studies are needed to unravel the various components contributing to supine SVI, HR, and thus CI (including patient selection). End-tilt SVI was lowest in the POTS group with a significantly higher SVI reduction compared to the two other groups. The lower SVI and the larger SVI reduction may be related to the aforementioned blood volume deficits, or the exaggerated HR increase by direct sympathetic stimulation of the sinus node, leading to a lower SVI. Although the SVI reduction was largest in POTS patients, due to the large increase in HR, the CI reduction was significantly less than the CI reduction in patients with a normal HR-BP response.

Figure 1 and Table 3 show the relations between HR and SVI. Even at rest, in the supine position, an inverse relation was observed between the supine HR and supine SVI in both patients and healthy controls (Figure 1). We described this relation between supine HR and SVI in a previous study [7]. Our data on the relationship between HR and SVI (and CI) are in line with a large Finnish cross-sectional study of healthy controls [50]. In the tertile of healthy controls with the highest supine HR, SVI was lowest and CI was highest, and vice versa. At end-tilt, a difference in SVI between POTS patients on one side and normal HR-BP patients and healthy controls on the other is observed (Figure 1). In POTS patients, there is no difference in SVI between patients with a low and high end-tilt HR: the slope between end-tilt HR and end-tilt SVI is not different from zero. This contrasts with the findings in normal HR-BP patients and healthy controls, where increasing end-tilt HRs are related to lower SVIs. This absent relation between end-tilt HR and SVI may hypothetically be caused by the fact that patients with the highest HR also have the highest catecholamines levels, which leads to an increased contractility, thereby reducing the SVI reduction. This remains to be proven.

There are many external factors that influence resting heart rate, stroke volume, and cardiac output, including external and body temperature, obesity, coffee, smoking, emotions, fitness status, age, medication, gender, thyroid abnormalities, sleep apnea, etc. Furthermore, the hemodynamics of the heart itself are influenced by sympathetic and vagal nerve activity, pre- and afterload [51], and cardiac contractile abnormalities. Despite these confounders, the present data show a clear relationship between HR and SVI, suggesting that the aforementioned factors play a minor role in this study.

The most important finding is that in patients with POTS, two different cardiovascular hemodynamic responses can be observed, as shown in Figure 1 and Figure 2 and in Table 4: in patients with POTS and a HR increase between 30–39 bpm, the decrease in SVI is larger than in POTS patients with a HR increase ≥ 40 bpm. The decrease in SVI of patients with a HR increase between 30–39 bpm follows the trend as observed in patients with a normal HR-BP response: from an increase of 0 bpm to an increase of 29 bpm during the tilt, there is a progressive SVI reduction. The hemodynamic data therefore suggest that, regardless of the underlying mechanisms in POTS [1,2], the patients with POTS and a limited HR increase between 30 and 40 bpm represent the right side of the spectrum of a SVI decrease due to venous pooling with a concomitant reflex HR increase. Whether there are hemodynamic differences between the proposed mechanisms (hypovolemia, deconditioning, inflammation like MCAS, auto-antibodies inhibiting vascular receptors, small fiber neuropathy, and hEDS with connective tissue laxity) needs to be determined in future research. Furthermore, POTS is a complex disease where involvement of the immune system, norepinephrine transporter deficiency, and impaired cerebral autoregulation also may play a role [52]. Finally, POTS has been shown to have a diurnal variation, with more patients showing POTS in the morning compared to the evening [53]. This again suggests a continuum of the HR-SVI relation between the patients with POTS and patients with a normal HR-BP response.

The other POTS patient group is those with a HR increase ≥40 bpm. In this group, the decrease in SVI and CI during the tilt is significantly less than in patients with a HR increase of 30–39 bpm. Possibly, this group of patients have a hyperadrenergic response with high norepinephrine levels while standing [41,54,55,56,57]. The increased norepinephrine levels may lead to an increased contractility, thereby augmenting SVI [58], and leading to a lesser SVI reduction in the present study. On the other hand, norepinephrine has a limited influence on heart rate [58]. There are multiple explanations for the excessive increase in HR in these POTS patients: a norepinephrine transporter dysfunction, resulting in a lower-than-normal norepinephrine clearance from the synaptic cleft [59], increased sympathetic firing with an increased cardiac norepinephrine spill-over [60], a baroreflex dysfunction with marked vagal impairment [61], an impairment of the renin-angiotensin system with a disturbed vasoconstrictor response [62], or an excessive sympathetic stimulation due to an increased venous pooling [63].

It needs to be stressed that, apart from a hypothesized hyperadrenergic response, the effects of the venous pooling are also operative in these POTS patients with a HR increase above 40 bpm. An unexpected finding was the transition from a large SVI reduction to a lesser SVI reduction around a HR increase of 40 bpm (Figure 1 and Figure 2). Possibly, in POTS patients with a HR increase above 40 bpm, the above-mentioned mechanisms play a more dominant role than in POTS patients with a limited HR increase. Another mechanism to consider is the presence/absence of hypocapnia as it may affect cardiac output [64]. These mechanisms need to be further explored. Finally, Yoshida et al. observed differences in HR and CI responses to the tilt in adolescent POTS patients [42]: patients with a high resting heart rate had a lower HR increase and a larger CI decrease compared to the POTS patients with a low resting heart rate. The authors hypothesized that compensatory mechanisms of sympathetic function, responsible for maintaining BP during standing, failed in the patient group with a high resting heart rate, probably because of exhaustion by the nearly maximum effort to generate sympathetic drive even in the supine position with low central blood volume [42]. In our POTS patient group supine HR was not different between patients with a HR increase of 30–39 bpm, versus those with a HR increase ≥40 bpm, but CI decrease was larger in the group with a HR increase of 30–39 bpm. However, their definitions of the two groups of POTS patients in the study of Yoshida et al. are different from the current definitions: the first group are adolescents with a HR increase ≥35 bpm, but the second group were patients with a standing HR ≥ 115 bpm and a HR increase < 35 bpm during active standing. The latter group, without a HR increase ≥35 or 40 bpm, is especially confusing and is out of line with the current definitions. This limits the interpretation and comparability of the data with our data.

Norepinephrine is clinically used for patient with low BP in the ICU. In a review, Lei et al. noted that the hyperadrenergic POTS was characterized by an SBP increase ≥10 mmHg in combination with a HR increase of at least 30 bpm [65]. However, the use of this SBP increase was not followed in subsequent publications. In our study, the SBP during tilt did not increase. The changes in SBP (and DBP) are variable, as reported in previous studies: in the study of Jacob et al. [66], NE rose to 900 but SBP decreased from 114 to 101 mmHg. In the study of Jacob et al. [67], NE rose to 840 while SBP did not significantly change. Garland et al. showed that the upright SBP were higher in patients with a high NE level while standing [41]. In the recent study of Okamoto et al., the SBP increase was only present in patients with a high muscle sympathetic nerve activity, but the differences compared to those patients with a low activity did not reach significance. In summary, the data on SBP increase during the tilt in hyperadrenergic POTS patient are variable and further studies are needed.

The main symptoms of orthostatic intolerance are on one hand related to the cerebral hypoperfusion (dizziness, memory problems, muscle weakness etc.), and on the other hand related to the sympathetic stimulation of the heart (palpitations, tremulousness, forceful beating of the heart, etc.) [68]. It is to be expected that patients with a HR above 40 bpm have more sympathetic stimulation-related symptoms than patients with a HR increase up to 40 bpm. On the other hand, a recent study of Angeli et al. showed that symptomatology cannot discriminate between what the authors called POTS phenotypes [69].

Finally, we previously showed that the reduction in cerebral blood flow during tilt testing is larger in POTS patients than in patients with a normal HR-BP response [29]. Whether there are differences in cerebral blood flow reduction between the two POTS patient groups studied here also needs to be established.

Our findings may have diagnostic and therapeutic implications. First, for the assessment of venous pooling vs. venous pooling plus a hyperadrenergic reaction, assessment of cardiac function as presented here may be needed. However, it is a time-consuming and costly procedure with its inherent learning curve. Finger plethysmography also presents data on stroke volume and cardiac output, but we previously demonstrated that this technique underestimates the changes during tilt testing [70]. Ideally, the discrimination between low and high heart rate increases during the tilt may be discriminative enough but that needs to be studied in future. Also, treatment strategies in ME/CFS patients may be based on hemodynamic findings, both in patients with POTS but also in patients with a normal HR and BP response. When venous pooling is predominantly present, patients may benefit from pharmacological and non-pharmacological interventions, like the use of increased water and salt intake, of compression garments, or of the prescription of fludrocortisone, desmopressin, midodrine, and pyridostigmine. In case of a hyperadrenergic reaction, beta-blockade or ivabradine may be preferable, whether in combination with blood volume increasing interventions or not. Also, this needs to be established in future randomized studies. Furthermore, the demonstration of objective abnormalities of circulatory dysfunction in ME/CFS contributes to the increasing body of evidence that ME/CFS is a chronic disease and not a psychological abnormality.

### Limitations

This is a single-centre observation and the findings of a differential hemodynamic response in POTS patients need to be replicated by others. In the present study we focused on cardiac function. Cardiac function is determined by three mechanisms: preload(filling), contractility, and afterload (systemic BP), which together determine stroke volume, and in combination with heart rate determine cardiac output. In the present study, systemic blood pressure does not significantly change, therefore the influence of afterload can be neglected. In both hypovolemic POTS and neuropathic POTS, venous pooling (larger SVI decrease) is larger than in the hypothesized hyperadrenergic POTS. Because we have no data on circulating blood volume as well as on small fiber neuropathy, we could not discriminate between the two, and data were taken together. It must be stressed that we also have no data on norepinephrine levels, nor data on immune markers that indicate an ongoing inflammatory process, which may contribute to the development of autonomic dysautonomia. These variables must be determined in future studies.

We acknowledge that referral bias by the general practitioner may have played a role, selectively referring patients with orthostatic symptoms. In our study we did not enroll those who were bedbound, as we elected not to expose those with more severe functional impairments to tilt testing. Patients with POTS were more severely diseased than patients with a normal HR-BP response. The independent effect of disease severity on the hemodynamic abnormalities also needs to be determined in future. The question is whether physical inactivity may have influenced the results. In one publication, we did not specifically study POTS patients but published the data of ME/CFS patients with a normal HR/BP during tilt testing and divided them into groups without deconditioning, with mild deconditioning, and with severe deconditioning. When categorizing the %VO_2_ peak as (a) absence of deconditioning, (b) mild, and (c) severe deconditioning [5], no significant differences were found in the three patient categories. Taken together, these findings provide no support for the hypothesis that deconditioning is a determining factor in the pathogenesis of orthostatic intolerance in ME/CFS [71]. Second, we compared the CBF reduction in ME/CFS patients with a normal HR/BP response during the tilt, with POTS, and with a delayed orthostatic hypotension. In all three groups, we compared the CBF reduction with the peak VO2. No relation was found between the CBF reduction and peak VO2 reductions. Importantly, in this study, 11% of the ME/CFS patients with POTS had no signs of deconditioning and 33% had mild deconditioning (see Table 4). Irrespective of the degree of deconditioning, all ME/CFS patients with POTS showed an abnormal CBF reduction during tilt table testing. If there had been a clear relationship between the degree of CBF reduction (objectively confirmed OI) and the degree of %VO_2_ peak reduction (objectively confirmed deconditioning), then this would have provided support for the hypothesis that exercise therapy would be beneficial for treating OI in ME/CFS. Instead, our data suggest that exercise therapy alone is unlikely to be effective in improving OI symptoms in this patient population, and that effective treatment of the orthostatic intolerance is more likely to lead to improved function. This holds true not only for ME/CFS patients with POTS, but also for ME/CFS patients with a normal HR and BP response and ME/CFS patients with dOH. As a clinical implication, therefore, exercise therapy is not likely to solve the problem of OI, at least in patients with ME/CFS [72]. Potential confounding factors like hypermobility, fibromyalgia, earlier corona infection, auto-immunity, mcas, on-going subclinical viral infection, small-fiber neuropathy, triggers other than a viral infection, etc. (see the list of comorbidities in Vernino et al. [2]) were not analyzed. Finally, other hemodynamic abnormalities in healthy controls with severe dehydration or in neurological patients with Parkinson disease or with MS were not studied here.

## 5. Conclusions

In patients with POTS, two different hemodynamic responses are present. In patients with a HR increase between 30–39 bpm, the decrease in SVI follows the trend as observed in patients with a normal HR-BP response: with increasing HR there is a progressive SVI reduction. In the other POTS patient group (those with a HR increase ≥40 bpm), the decrease in SVI and CI during the tilt is significantly less than in patients with a HR increase of 30–39 bpm. We speculate that this group of patients has a hyperadrenergic response to upright posture. This may have therapeutic implications where POTS patients with a limited HR increase may benefit from interventions aiming at increasing the circulating blood volume, while patients with a high heart rate may preferably treated with beta-blockers or ivabradine. This needs to be prospectively assessed. Finally, there is growing evidence that ME/CFS and long-COVID patients share the same clinical and hemodynamic abnormalities [73,74,75]. Therefore, our findings and potentially the therapeutic implications may also be applicable to the large long-COVID population

## Figures and Tables

**Table 1:** Baseline characteristics of subjects undergoing tilt testing: ME/CFS patients with postural orthostatic tachycardia syndrome (POTS) or with a normal heart rate and blood pressure response and healthy controls with a normal heart rate and blood pressure response.

	Group 1 ME/CFS with POTS (*n* = 233)	Group 2 ME/CFS with Norm HR-BP (*n* = 507)	Group 3 Healthy Controls (*n* = 47)	One-Way Welch ANOVA/Kruskal–Wallis/Mann–Whitney U/Chi-Square: *p*-Value
Male/female *	26/207 (11/89%)	84/423 (17/83%)	10/37 (21/79%)	*p* = 0.077
Age (years) ∞	34 (10)	41 (12)	38 (14)	F (2, 786) = 29.179; *p* < 0.0001; 1 vs. 2 *p* < 0.001
Height (cm) ∞	173 (8)	171 (8)	173 (7)	F (2, 786) = 6.284; *p* = 0.002; 1 vs. 2 *p* = 0.002
Weight (kg) #	67 (59–78)	70 (62–81)	71 (62–81)	X_2_ = 7.380; *p* = 0.025
BMI (kg/m^2^) #	22.2 (20.3–25.2)	23.9 (21.2–27.6)	24.2 (21.3–27.3)	X_2_ = 20.309; *p* < 0.001; 1 vs. 2 *p* < 0.001
BSA (m^2^)	1.82 (0.19)	1.84 (0.20)	1.86 (0.18)	F (2, 786) = 0.5883; *p* = 0.59
Disease duration (years) ##	9 (4–15)	12 (6–20)	NA	*p* < 0.001
Disease severity ^®^: mild/moderate/severe	56/117/60 (24/50/26%)	171/260/76 (34/51/15%)	NA	*p* < 0.001

BMI: body mass index: BSA: body surface area (duBois formula); HC: healthy controls; Norm HR-BP: normal heart rate and blood pressure response during tilt testing; ME/CFS: myalgic encephalomyelitis/chronic fatigue syndrome; POTS: postural orthostatic tachycardia syndrome; ∞: one-way Welch ANOVA with Tukey post hoc test; *: Chi-square analysis, #: Median (IQR), Kruskal–Wallis test with Dunn post hoc test, ##: Mann–Whitney U test; ^®^: disease severity according to the ICC criteria [27].

**Table 2:** Hemodynamic data of subjects undergoing tilt testing: ME/CFS patients with postural orthostatic tachycardia syndrome (POTS) or with a normal heart rate and blood pressure response and healthy controls with a normal heart rate and blood pressure response.

	Group 1 ME/CFS with POTS (*n* = 233)	Group 2 ME/CFS with Norm HR-BP (*n* = 507)	Group 3 Healthy Controls (*n* = 47)	One-Way Welch ANOVA/Kruskal–Wallis: *p*-Value
supine HR (bpm) ∞	74 (12)	69 (11)	62 (9)	F (2, 786) = 29.179; *p* < 0.001; 1 vs. 2 < 0.001; 1 vs. 3 *p* < 0.001; 2 vs. 3 *p* < 0.001
end-tilt HR (bpm) #	111 (103–122)	83 (75–92)	79 (65–87)	X_2_ = 373.0; *p* < 0.001; 1 vs. 2 *p* < 0.001; 1 vs. 3 *p* < 0.001
delta HR (bpm) ∞	39 (9)	15 (8)	15 (8)	F (2, 786) = 715; *p* < 0.001; 1 vs. 2 *p* < 0.001; 1 vs. 3 *p* < 0.001
supine SVI (mL/m^2^) ∞	37 (6)	38 (6)	37 (5)	F (2, 786) = 0.7029; *p* = 0.352
end-tilt SVI (mL/m^2^) #	19 (17–21)	22 (20–25)	26 (24–31)	X_2_ = 155.5; *p* < 0.001; 1 vs. 2 *p* < 0.001; 1 vs. 3 *p* < 0.001; 2 vs. 3 *p* < 0.001
delta SVI (mL/m^2^) ∞	−18 (4)	−15 (4)	−10 (3)	F (2, 786) = 85.467; *p* < 0.001; 1 vs. 2 *p* < 0.001; 1 vs. 3 *p* < 0.001; 2 vs. 3 *p* < 0.001
supine CI (L/min/m^2^) ∞	2.73 (0.49)	2.58 (0.46)	2.28 (0.33)	F (2, 786) = 20.54; *p* < 0.001; 1 vs. 2 *p* = 0.002; 1 vs. 3 *p* < 0.001; 2 vs. 3 *p* < 0.001
end-tilt CI (L/min/m^2^) #	2.13 (1.79–2.52)	1.87 (1.65–2.11)	2.04 (1.88–2.26)	X_2_ = 54.05; *p* < 0.001; 1 vs. 2 *p* < 0.001; 2 vs. 3 *p* = 0.001
delta CI (L/min/m^2^) ∞	−0.53 (0.41)	−0.67 (0.25)	−0.20 (0.16)	F (2, 786) = 61.94; *p* < 0.001; 1 vs. 2 *p* < 0.001; 1 vs. 3 *p* < 0.001; 2 vs. 3 *p* < 0.001
supine SBP (mmHg) ∞	132 (15)	137 (18)	135 (16)	F (2, 786) = 6.922; *p* = 0.001; 1 vs. 2 *p* < 0.001
end-tilt SBP (mmHg) ∞	127 (18)	134 (18)	126 (15)	F (2, 786) = 13.38; *p* < 0.001; 1 vs. 2 *p* < 0.001; 2 vs. 3 *p* = 0.009
supine DBP (mmHg) ∞	80 (10)	81 (11)	79 (8)	F (2, 786) = 1.243; *p* = 0.289
end-tilt DBP (mmHg) ∞	88 (14)	87 (13)	81 (8)	F (2, 786) = 5.57; *p* = 0.004 2 vs. 3 =0.004; 1 vs. 3 *p* = 0.003

CI: cardiac index; delta CI decrease: absolute reduction in cardiac index end-tilt vs. supine; delta HR: increase in HR (end-tilt minus supine); DBP: diastolic blood pressure; HR: heart rate (as measured by echocardiography); ME/CFS: myalgic encephalomyelitis/chronic fatigue syndrome; SBP: systolic blood pressure; SVI: stroke volume index; delta SVI decrease: absolute reduction in stroke volume index end-tilt vs. supine; ∞: one-way Welch ANOVA with Tukey post hoc test; #: median (IRQ), Kruskal–Wallis test with Dunn post hoc test.

**Table 3:** Linear regression analysis of supine, end-tilt, and change in HR, SVI, and CI data in patients with postural orthostatic tachycardia syndrome, in patients with a normal HR-BP response during the tilt, and in healthy controls with a normal heart rate and blood pressure during the tilt.

Group	SVI (Y) vs. HR (x) *	Linear Regression Line	R^2^	*p*-Value
Group 1, ME/CFS with POTS	SVI supine	Y = −0.185x + 5028	0.157	*p* < 0.001
	SVI end-tilt	Y = 0.005x + 18.6	0.000	*p* = 0.784
	Delta SVI	Y = 0.092x − 21.6	0.036	*p* = 0.004
Group 2, ME/CFS with normal HR-BP response	SVI supine	Y = −0.220x + 52.9	0.159	*p* < 0.001
	SVI end-tilt	Y = −0.154x + 35.9	0.192	*p* < 0.001
	Delta SVI	Y = −0.308x −10.1	0.273	*p* < 0.001
Group 3, healthy controls	SVI supine	Y = −0.284x + 54.8	0.253	*p* < 0.001
	SVI end-tilt	Y = −0.232x + 45.3	0.390	*p* < 0.001
	Delta SVI	Y = −0.273x − 5.6	0.368	*p* < 0.001

BP: blood pressure; HR: heart rate; POTS: postural orthostatic tachycardia syndrome; SVI: stroke volume index. ME/CFS: myalgic encephalomyelitis/chronic fatigue syndrome; *: supine or end-tilt, or changes (delta: end-tilt minus supine) in parameters, where appropriate.

**Table 4:** Hemodynamic results of ME/CFS patients with postural orthostatic tachycardia syndrome (POTS) with a heart rate increase of 30–39 bpm and ME/CFS patients with POTS with a heart rate increase ≥40 bpm.

	Group 2 POTS with HR Increase of 30–39 bpm at End-Tilt (*n*= 145)	Group 3 POTS with HR Increase of ≥40 bpm at End-Tilt (*n* = 88)	*t* Test/Mann–Whitney U Test: *p*-Value
supine HR (bpm)	74 (12)	75 (12)	*p* = 0.48
end-tilt HR (bpm)	105 (98–114)	121 (113–132)	*p* < 0.001
delta HR (bpm)	33 (3)	48 (7)	*p* < 0.001
supine SVI (mL/m^2^)	38 (5)	36 (7)	*p* = 0.119
end-tilt SVI (mL/m^2^)	18 (17–20)	20 (17–23)	*p* = 0.005
delta SVI (mL/m^2^)	−19 (4)	−16 (5)	*p* < 0.001
supine CI (L/min/m^2^)	2.75 (0.49)	2.69 (0.48)	*p* = 0.366
end-tilt CI (L/min/m^2^)	1.93 (1.71–2.17)	2.53 (2.21–2.90)	*p* < 0.001
delta CI (L/min/m ^2^)	−0.77 (0.24)	−0.12 (0.28)	*p* < 0.001
supine SBP (mmHg)	132 (15)	133 (15)	*p* = 0.647
end-tilt SBP (mmHg)	128 (18)	127 (19)	*p* = 0.874
supine DBP (mmHg)	80 (11)	80 (8)	*p* = 0.672
end-tilt DBP (mmHg)	89 (14)	87 (13)	*p* = 0.314

SBP: systolic blood pressure; DBP: diastolic blood pressure HR: heart rate; POTS: postural orthostatic tachycardia syndrome; SVI: stroke volume index. CI: cardiac index.

## Data Availability

Not applicable.
